# Protist community sites and structure under two barn management systems at a commercial dairy

**DOI:** 10.3389/frmbi.2026.1803341

**Published:** 2026-05-14

**Authors:** Tawni L. Crippen, Dongmin Kim, Sonja L. Swiger, Robin C. Anderson

**Affiliations:** 1 Southern Plains Agricultural Research Center, Agricultural Research Service, US Department of Agriculture, College Station, TX, United States; 2 Department of Entomology, Texas A & M University, College Station, TX, United States; 3 Entomology Extension, Texas AgriLife, Texas A & M University, College Station, TX, United States

**Keywords:** bovine, Diptera, microbiome, pathogen, Protista, shotgun metagenomics

## Abstract

**Introduction:**

Investigations into the location and load of protists in the environment arounddairies are scarce but are essential to maintaining the health of livestock.Moreover, the design of dairy barns has fluctuated over the decades to maximizecattle health and milk production without regard to influences on environmentalmicrobiomes. Beyond cost, the major emphasis of barn design is the managementof appropriate temperature and comfort for cattle. However, there havebeen no corresponding investigations into whether these design changes affect protist communities within barns.

**Methods:**

In this study, community shotgun metagenomic analysis was used to define the spatial composition and relative abundance of protist communities from 118 samples of manure, lagoons, troughs, and house and stable flies at a commercial dairy implementing two free-stall management systems: flow-through and cross-vent. Sequence reads were mapped to the CosmosID database. Viability was not assessed; therefore, results reflect DNA detection only not viability or disease occurrence.

**Results:**

The protist composition differed significantly between dairy components. Ecological findings showed that troughs and lagoons harbored high protist diversity, including the possible pathogen Neobalantidium coli and potential carriers Paramecium biaurelia and Acanthamoeba. Manure had the lowest protist diversity. Stable flies carried more protist taxa than house flies. Both fly species uniquely carried the non-pathogenic alveolate parasite Hammondia hammondi. The water mold plant pathogen Pseudoperonospora cubensis was identified in all sample types. Of the total relative abundance of protists, 2.10% were amoebas, 7.63% alveolate parasites, 62.71% water molds, 23.31% ciliates, 1.74% foraminifera, and 2.50% diatoms.

**Discussion:**

These results describe preliminary spatial overlaps and possible avenues of dissemination, providing a basis for assessing appropriate management systems and identifying protist reservoir sites within dairy operations.

## Highlights

- Protist communities were contrasted within dairy barns employing two management systems.
- Protist diversity differed significantly among components within management systems.
- Troughs and lagoons harbored the greatest number of protist species; manure harbored the fewest.
- A plant pathogen was the most prevalent protist detected in the samples.
- Flies primarily carried a non-pathogenic alveolate parasite and the plant pathogen.

## 1 Introduction

According to the National Agricultural Statistics Service (NASS), the number of milk cows in the United States as of March 21, 2025, was 9,410,000 (USDA, 2025). The design of dairy barns has changed significantly over the decades to maximize dairy cattle health and, correspondingly, milk production. One element of these design changes is the maintenance of comfortable temperatures and air quality within barns to reduce cattle stress (Beaupied et al., 2022). However, this has occurred without corresponding investigations into whether these changes affect the distribution and load of environmental microbial communities within these facilities. In this study we focus on protists, particularly those that may be pathogenic, because of their importance to dairy operations. While barn management systems are purposely designed to regulate temperature and humidity within the facilities and maintain cattle well-being, these factors may also affect the incidence of protists within the dairy environment.

Precise management of the dairy environment is required to help cattle remain in a thermoneutral zone that maximizes milk production and animal health by maintaining consistent air quality and exchange rates (Berman, 2005). Different stall management systems are employed in dairies across the country to accomplish this goal (Lobeck et al., 2011). In conventional flow-through, free stall-designed facilities, cattle are not restricted to a single stall and have the opportunity to move freely around the enclosure and interact with others in the herd (Tyson et al., 2022). Cross-vent barns have a similar free-stall arrangement; however, they have baffles that hang from the ceilings to redirect the air at a greater velocity into the stalls. These systems provide ventilation advantages by reducing cross-sectional areas within the barn and thereby increasing airflow at the animal level to maintain proper thermoneutral zones (Wang et al., 2018). The exhaust fans on one exterior wall and air intake (via evaporative panels) on the opposing wall help to regulate humidity and temperature; this cross-vent style also reduces the indoor concentration of harmful gases (Wolf and McBride, 2018). Within these different management systems, various microorganisms have the opportunity to survive and be transferred and exchanged between environmental elements and surfaces (Bradford et al., 2013; Matos et al., 2025).

Many of the microbes associated with cattle are essential for their health, but some are detrimental to the cattle, their milk production, and the humans caring for them. Some pathogens are passed to humans via dairy products, while others are transmitted through contact during preharvest rearing and production (FDA, 2012). The prevalence of illnesses from dairy products is, however, low. Only about 2% of human illnesses were reported as associated with dairy products in 2017 by the Centers for Disease Control and Prevention (CDC) and were usually attributed to unpasteurized products (CDC, 2019). Of additional concern are the mortality and morbidity of dairy cattle and the subsequent economic and genetic losses to producers. According to the National Animal Health Monitoring System’s (NAHMS) last survey (2014), the mortality rate for dairy cattle in the US was 5.6%, and a high percentage of those deaths were from scours in pre-weaned heifers (USDA, 2021). Various bacterial, viral, and protist pathogens are common causes of scours in cattle (Cho and Yoon, 2014).

Protists are an understudied group of microorganisms encompassing a highly diverse group of primarily unicellular eukaryotes such as protozoa, algae, slime molds, and diatoms. They do not fit neatly into the Animalia, Plantae, or Fungi kingdoms (Corliss, 2002; Adl et al., 2019). Although they are found globally in a variety of habitats, their diversity and dispersal are difficult to estimate due to undersampling and their propensity to exist in nature in dormant (cystic) stages (Foissner, 1999, 2008). Protists prefer aquatic or moist terrestrial environments, exist free-living or in aggregates, and may also be parasitic or beneficial mutualistic symbionts (Foissner, 2008). They are foundational within the food chain and serve many vital functions, such as nutrient recycling of nitrogen and carbon, and contribute to soil fertility, plant growth, and general ecosystem health (Chandarana and Amaresan, 2022; Nguyen et al., 2023). In animal production facilities, the intestinal microbiome of livestock is the principal source of microorganisms in excrement, although other abiotic and biotic sources also contribute (Abdugheni et al., 2023). Of the microbial components in livestock manure, approximately 5% were found to be protozoa and fungi (Abdugheni et al., 2023). In the subsequent wastewater lagoon, where excrement is washed, protists can reduce the bacterial load and metabolize organic solids and chemicals into usable nutrients that improve the quality of wastewater (Papadimitriou et al., 2007; Saleem and Moe, 2014; Tiwari and Marella, 2019). However, there are also protists that are detrimental and cause plant and animal diseases (Niculescu, 2014; Rossi et al., 2024). Studies of these detrimental protists in dairy animals have received the bulk of scientific attention. There is a scarcity of research on the environmental sources and reservoirs of protists within dairy facilities. This study begins to address some of that deficiency.

We investigated whether the management style differences (cross-vent versus conventional flow-through barns) could affect the community structure and location of protists, which are also affected by the temperature and moisture content of their environment (Glaser et al., 2015; Li et al., 2023). The sampling design included manure, lagoons, and troughs associated with the two barn management systems. Additionally, we sampled flies, which are common microbial vectors at dairies and have been previously shown to carry bacteria at commercial dairies (Crippen et al., 2023). The two most common species sampled, house flies (*Musca domestica* L.) and stable flies (*Stomoxys calcitrants* L.), represent different Diptera feeding systems: sponge and blood feeders, respectively (Renčínová et al., 2021). While all protists identified are described here, the identification of pathogenic species was one of our focuses. We expected more communities of protists in the CV barn management systems because CV barns maintain more consistent temperatures and humidity conditions conducive to protist habitat. The purpose of this study was to 1) investigate the occurrence of protists within specific environmental elements at a working dairy implementing two different barn management systems, 2) determine where possible pathogenic protists reside, and 3) discern the overlap of species between the different elements sampled and thus possible pathways of pathogenic spread. The protist community composition was measured by whole-genome sequencing to determine diversity and abundance at various environmental locations within the dairy that could serve as sources, disseminators, and reservoirs for protists. We expected higher richness in the composition of protist in the wet areas of the dairy and overlap of species between the connected compartments.

## 2 Materials and methods

### 2.1 Site

The dairy is located in North Texas on generally sandy to loamy soil supporting tall- to short-grass mixed prairie. During the summer (June-August) of 2018, conditions were dry, with erratic rainfall (average 0.012 cm precipitation). Temperatures were high, with a mean day and night temperature of 34.2 and 28.5 °C, respectively (https://world-weather.info/). The operation was a large dairy, maintaining ≥ 500 head of Holstein dairy cattle, bred using artificial insemination. Heifers were in pastures and dry lots; dry cows and infirm cows were in free-stall flow-through (FT) barns. The lactating cows, which were milked twice a day, were in side-by-side open stalls (1.2 x 2.8 m) in a cross-vent (CV) barn. Cattle were fed forage-based total mixed rations consisting of corn silage, sorghum, and coastal grass twice daily. The CV barn contained a feedline water soaker system with baffle curtains that directed airflow directly over the cows, with automated lighting (LED lights 18:6 h L:D). Barns were manually cleaned using a vacuum and then pressure-washed. Floor surfaces in the cow lanes were sloped to allow tank flushing and/or manual cleaning by pressure washer, directing waste into the lagoon system. The milking parlor and CV barn wash drainage combined into multiple waterways leading to three sequential, uncovered, anaerobic/facultative, naturally aerated lagoons. The FT manure waste was manually collected and dumped into the first of another three-lagoon sequential system connected by waterways.

This study represents a single-site dairy evaluated at a single time point, which limits generalization to other agroecosystems. This dairy was chosen because both management systems were implemented at the same location and therefore would inherently exclude the influence of climate and landscape differences. Additionally, the cattle were fed the same feed and underwent the same preventative health regimen. Sampling of the cattle themselves was not feasible due to the potential for animal stress and subsequent effects on milk production. Sampling of milk prior to pasteurization was not allowed by the producer, as this was a working commercial dairy operating under strict health, safety, and hygiene protocols.

### 2.2 Sampling design and DNA extraction

Common elements within both management systems at the dairy (manure, lagoons, and troughs) were chosen for sampling. Further, house flies representing sponge feeders and stable flies representing biting blood-feeding insects were sampled. This allowed us to determine whether flies were purveyors of protists within the dairy and whether their two nutritional approaches affected their protist communities. It should be noted that the sampling design consisted of composite samples to gain broader coverage across the large dairy area, while maintaining a feasible study size both economically and logistically. Thus, the design does not provide the spatial resolution required for detailing precise protist locations but instead captures a broader site-level representation.

Sterile specimen cups (100 mL) were used to aseptically collect samples from the different elements within the CV and FT barns at the dairy (CV manure, CV lagoon, CV trough, CV house fly, CV stable fly, FT manure, FT lagoon, FT trough, FT house fly, and FT stable fly) in June 2018. To avoid interference with dairy operations and minimize stress to the cattle, no samples were taken directly from the animals or the milk. In the CV and FT barns, 100 samples, consisting of approximately 10 g of manure per sample, were collected in each barn from the stalls and alleyways. After DNA extraction (see below), composites were made by combining samples into groups of five, resulting in a final count of 20 CV manure and 20 FT manure samples. Samples of 100 mL mixtures of particulate and aqueous material from lagoons along the water-soil interface lagoon were aseptically collected along the circumference of the shoreline from the interconnected CV lagoons, and the same procedure was followed for the interconnected FT lagoons. Although the multiple lagoon samples were taken from independent sites along the edges of the multi-lagoon systems, the lagoons were interconnected, resulting in pseudo-replication, despite differing conditions among sequential lagoons. This resulted in 20 samples from each of the CV and FT lagoon systems. The manure and lagoon DNA were extracted from an aliquot of 0.4 g of manure or lagoon using the MP FastDNA Spin Kit for Feces (MP Biomedicals, Irvine, CA) per the manufacturer’s instructions.

A 500 mL aliquot of trough water was collected from 10 troughs in each CV and FT management systems. The troughs were filled from the same initial water source; however, their placement within the barns allowed for independent interaction with cattle in each alleyway. Water samples were first vacuum-filtered through sterile bottle-top 0.22 µm filters (Corning Inc., Glendale, AZ). The filters were used for DNA extraction. Insects were collected by sweep-netting the alleyways beside the stalls. The sampled insects were transported to the laboratory in coolers and aseptically sorted by species. Only house and stable flies were retained because these flies represent two different feeding approaches. The house fly feeds on a wide range of food sources by expelling salivary digestive enzymes to ensure that the food source is liquified prior to sponging. The stable fly is a hematophagous biting fly adapted for piercing the skin and feeding on blood. For DNA extraction, each sample contained 10 flies, resulting in CV house fly (n = 5), FT house fly (n = 5), CV stable fly (n = 5), and FT stable fly (n = 3) samples. The flies were not surface-disinfected, and the entire fly was utilized, so both internally and externally carried microbes were included. The flies were pooled to capture broader site representation and to ensure sufficient DNA quality and quantity for sequencing. The DNA from the flies and trough filters was extracted by an organic phenol-chloroform method. This involved cell lysis using 0.1mm zirconium beads (OPS diagnostics, Lebanon, NJ) in Carlson lysis buffer (Fisher Scientific, Fair Lawn, NJ), followed by disruption at 6m/s for 40 s on a FastPrep-24 homogenizer (MP Biomedicals, Irvine, CA) and incubation for 1 h at 37 °C with 15 mg/ml lysozyme (Fisher Scientific). This was followed by incubation for 16 h at 65 °C with 10% sodium dodecyl sulfate (Fisher Scientific) and 100 μg/ml proteinase K (Fisher Scientific). The top layer was retained after centrifugation for 1 min at 17,000 x g. An equal volume of 25:24:1 phenol/chloroform/isoamyl alcohol (Sigma-Aldrich, St. louis, MO) was added and centrifuged for 6 min at 17,000 x g at 4 °C. The aqueous layer was retained, and 100 μg/ml ribonuclease A (Amresco Inc., Solon, OH) was added and incubated for 30 min at 37 °C. An equal volume of 24:1 chloroform/isoamyl alcohol (Sigma-Aldrich) was added and centrifuged for 6 min at 17,000 x g at 4 °C, and the aqueous layer was retained. This step was repeated. The DNA was precipitated with isopropyl alcohol and centrifuged for 25 min at 17000 x g at 4 °C. The pellet was washed with 70% ethanol, followed by centrifugation for 25 min at 17000 x g at 4 °C. An in-house microbial control mix was included throughout the entire extraction and sequencing processes to test identification accuracy analyses. Upon analysis, no protist contamination was detected. The final number of samples sequenced from each element was as follows: CV manure (20), CV lagoon (20), CV trough (10), CV house fly (5), CV stable fly (5), FT manure (20), FT lagoon (20), FT trough (10), FT house fly (5), and FT stable fly (3).

### 2.3 Sequencing and bioinformatics analyses

The total number of dairy samples after DNA extraction was 118, which were normalized to 50 ng/µL using a microvolume spectrophotometer (DeNovix Inc, Wilmington, DE) and then stored at -20 °C until sequencing. Whole-genome shotgun metagenomic sequencing was performed on the DNA using the CosmosID algorithms (CosmosID Inc., Germantown, MD). *Allobacillus halotolerans* and *Imtechella halotolerans* K1 were included as bacterial run controls for sequencing. The DNA was quantified using a Qubit fluorometer (Invitrogen Co., Carlsbad, CA). Nextera XT DNA Library Preparation Kit and Nextera Index Kit (Illumina, Inc., San Diego, CA) were used to prepare the DNA libraries using a total DNA input of 1 ng. Genomic DNA was fragmented using a proportional amount of Illumina Nextera XT fragmentation enzyme (Illumina). Combinatorial dual indexes were added to each sample, followed by 12 cycles of PCR to construct libraries. AMpure magnetic beads (Beckman Coulter, Inc., Brea, CA) were used to purify DNA libraries, which were eluted in QIAGEN EB buffer (Qiagen, Inc., Redwood City, CA) and quantified again using a Qubit fluorometer and Qubit™ dsDNA HS Assay Kit (Invitrogen). Libraries were sequenced on an Illumina HiSeq platform (2 x 250 bp; Illumina, Inc.), and the raw reads (2,172,237,723 total reads) were processed by MultiQC (v1.11, Sequera Labs, S.L., Barcelona, Spain) to verify that read quality met threshold criteria (Phred score > 20) without excessive adapter content. The median number of reads per sample was 18,408,794.26 reads. The 118 samples were processed for quality control by performing general statistics on sequencing efficacy, which showed an average GC content of 54.41% and 151 bp length (11.39% of modules failed). The sequencing depth of these samples ranged from 8.7 to 32.3 million reads, supporting sensitive and comprehensive profiling. One sample (FT trough) had low read depth (< 8M) and was removed from the analyses. Abundances were calculated as normalized relative read proportions; relative abundance metrics were depth-normalized to account for variation across samples. Taxa were considered present if relative abundance exceeded 0.01% and quality filters (k-mer/coverage) were met.

Metagenomic samples were uploaded to the Cosmos-Hub (www.cosmos-hub.com) platform for taxonomic profiling (Colwell et al., 2018; Hasan et al., 2020; Colwell et al., 2022). The processing of the raw read data was performed in collaboration with CosmosID to map the reads using their custom-curated bacterial, fungal, viral, and protist genomic database. Classification methods utilized a high-performance data-mining k-mer-based algorithm that disambiguated the millions of short sequence reads into discrete genomes engendering the particular sequences. Positive and negative internal controls were included to ensure that these generated the expected results. Identification was based on whole genomes referenced in the NCBI-RefSeq/GenBank/WGS/SRA/™ database (Franzosa et al., 2018). Taxonomic classification methods were performed according to the CosmosID continuously curated databases of reference genomes (Hasan et al., 2014; Lax et al., 2014; Ponnusamy et al., 2016). Species calls required multiple uniquely mapping, species-specific k-mers with a consistent depth pattern and a minimum relative abundance of 0.01% at the species level. This 0.01% cutoff is an empirical threshold used in CosmosID profiling to balance the detection of low-abundance taxa against spurious noise, supported by internal benchmarking on mock communities. Abundance scores were calculated by translation of the fine-grain composite k-mer statistics, coverage depth estimation, and genome size information. The relative abundance was calculated by dividing the counts for each taxon by the sum per sample for downstream comparative analysis or differential abundance analysis.

*Pseudoperonospora cubensis* is known as a water mold; however, this species does not classify as a fungus and is included in this analysis as a protist per the reference database used by CosmosID taxonomic profiling (see **Supplementary Material** for classification).

### 2.4 Statistics

The diversity analyses, principal coordinate analysis (PCoA), and linear discriminant analysis effect size (LEfSe) of the dairy component protist communities were computed using the CosmosID-HUB Microbiome application (https://app.cosmosid.com/samples). Alpha diversity metrics were calculated according to their original, published statistical formulations using normalized count data (Chao, 1984; 1987). Comparisons between component alpha diversity indeces were performed using the Wilcoxon rank-sum test based on abundance scores. The alpha diversity analysis used normalized count data, while the beta diversity analysis used relative abundance. No normalization/transformation was performed beforehand. The beta diversity index was assessed using Bray-Curtis dissimilarity and PREMANOVA analysis of relative abundances with 999 permutations in the CosmosID-HUB platform, at a significance value of *p* < 0.001; no correction for multiple comparisons or checks on dispersion homogeneity were applied (Jaccard, 1912; Bray and Curtis, 1957). As implemented, diversity index calculations do not assume underlying data distributions; real-world microbiome datasets are typically non-parametric (Zhou et al., 2023). The CosmosID framework does not incorporate the specialized partitioning methodologies proposed by Baselga (2010) or Podani and Schmera (2011). However, these frameworks, which respectively partition beta diversity into turnover/nestedness (Baselga) and replacement/richness difference (Podani & Schmera), conceptually overlap with the classic diversity measures in interpreting compositional differences across communities. No data transformations were applied.

The PCoA was displayed using JMP® 15.1.0 (SAS Institute Inc., Cary, NC). Indicator species analysis using LEfSe was used to characterize the species differences between two or more communities in the samples and the taxa that best discriminate their differences, based on percent relative abundances (Segata et al., 2011). The linear discriminant analysis score (LDA) was obtained by computing the logarithm (base 10) of this value and indicated the effect size for species biomarkers found to be discriminative among each cohort, using a *p*-value of *p* < 0.001 for the comparative factorial Kruskal-Wallis test. Venn comparisons of the individual elements (CV manure, CV lagoon, CV trough, CV house fly, CV stable fly, FT manure, FT lagoon, FT trough, FT house fly, and FT stable fly), in addition to the combined management style (CV and FT) and components (manure, lagoon, trough, house fly, and stable fly) of the dairy protist communities, were performed using the InteractiVenn (http://www.interactivenn.net/) application (Heberle et al., 2015). Only organisms identified to at least the genus level were included in the analysis. The smaller number of FT stable fly samples limits conclusions regarding comparative differences and overlaps for this component.

## 3 Results

**Figure 1:**
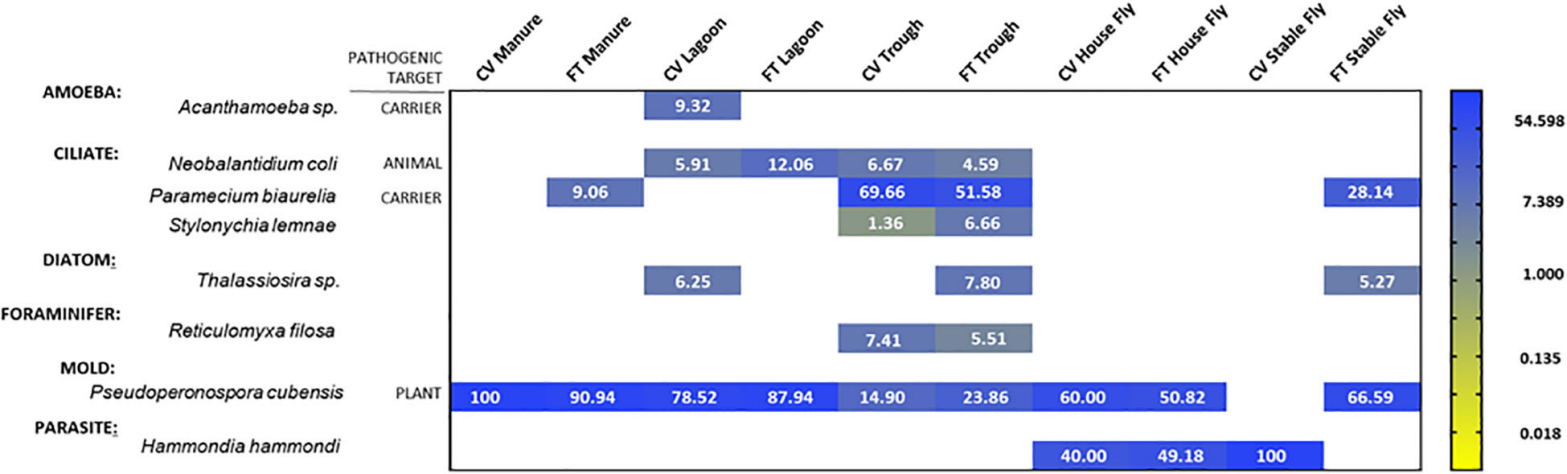
Heat map of the percent relative abundance of the protists identified to the species level within the components (manure, lagoon, trough, house fly and stable fly) of the cross-vent (CV) and flow-through (FT) free-stall management systems. For natural log transformation, “0” values were assigned the value of “0.001”.

The dairy components selected for sampling represent major habitat differences. Troughs consistof water with limited organic sediments in comparison to other components sampled, whereas manure is higher in organic material but has less moisture than troughs. Lagoons are primarily manure mixed with other dairy materials, such as wash water, urine, bedding materials, and feed. They are anaerobic and exhibit very distinctive conditions, such as high organic content, near neutral pH, and aerobic and temperature conditions that vary with depth (Bella and Rao, 2023). These components, along with the flies, are connected through access to cattle and cattle behavior and therefore offer possible pathways of microbial spread. The seasonality of the presence of fly species can be difficult to predict in Texas. Unfortunately, the number of stable flies present at the time of sampling was low, which weakened comparative analyses with this component. The number of protist species found in each element (n) was as follows: CV manure (1), CV lagoon (4), CV trough (5), CV house fly (2), CV stable fly (2), FT manure (2), FT lagoon (2), FT trough (6), FT house fly (2), and FT stable fly (3) (**Figure 1**). A list of the protist species identified within a specified single component or shared by a collective of components when CV and FT free-stall management systems were combined is supplied (**Supplementary Table 1**).

Of the 118 samples taken, 71 were positive for protists. The species identified belonged to three protist phyla. From the phylum Apicomplexa, six were positive for the obligate parasite *Hammondia hammondi*. From the phylum Bacillariophyta, eight were positive for the photosynthetic diatom *Thalassiosira* sp. From the phylum Eukaryota, 66 samples were positive for the water mold *Pseudoperonospora cubensis*, 19 for the free-living *Paramecium biaurelia*, 4 for the parasitic ciliate *Neobalantidium coli*, 2 for the free-living and occasionally parasitic *Acanthamoeba* sp., 9 for the free-living *Reticulomyxa filosa*, and 9 for the free-living *Stylonychia lemnae*. Their percent relative abundances within each element are presented in **Figure 1**.

### 3.1 Diversity comparisons

**Figure 2:**
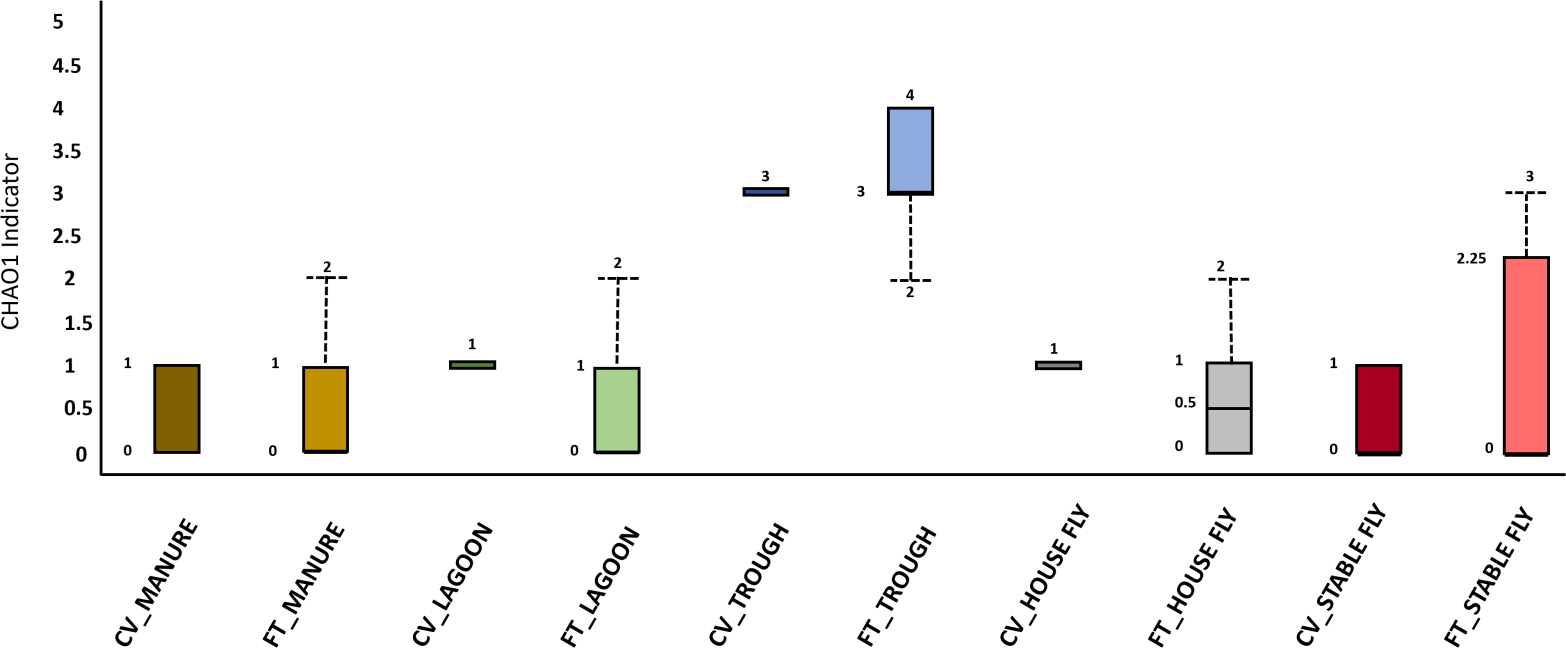
**Figure 1**. Alpha diversity graphic: Box and whisker diagram of CHAO1 diversity results of the protist communities of the elements within the dairy components (manure, lagoons, troughs, stable fly, and house fly), separated into results from both dairy management systems: cross-vent (CV) and flow-through (FT) free-stall systems.

The differences among individual elements and the combined management-style components at the dairy were examined by determining community diversity. The sampling design included composite samples to broaden community-level representation of the large dairy. However, the pooling of samples may mask the individual variation and heterogeneity of intra- and inter-group variance for diversity analyses. Thus, it would reflect the average density of species rather than the presence/absence of species in individual spatial sites. Alpha diversity indeces (CHAO1) were used to compare the number of protist species within each element (**Figure 2**) within the CV or FT management systems. No significant difference (*p* ≤ 0.001) in richness was observed between CV and FT components. However, differences were observed when species communities of individual elements were compared within each management system.

#### 3.1.1 Beta diversity analyses

**Figure 3:**
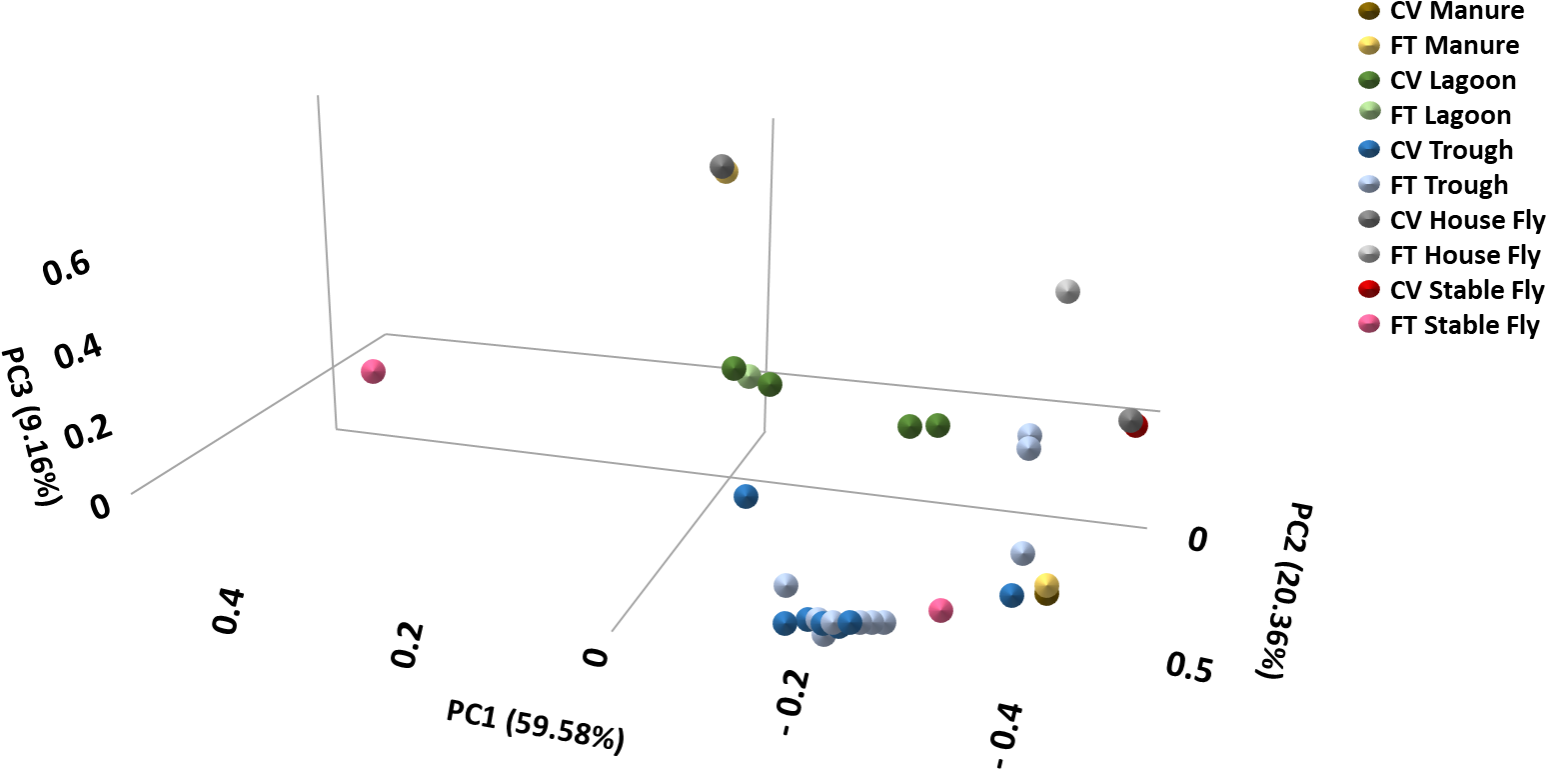
Principal coordinate analysis scatter plot using Bray-Curtis dissimilarity values of relative abundances of protist lineages within the dairy communities. Samples were collected from the components listed within the cross-vent (CV) and flow-through (FT) management areas.

The beta diversity metric was used to analyze the differences in species composition among dairy samples (**Table 1**). The principal coordinate analysis plot using Bray-Curtis dissimilarity values of relative abundances allowed the visualization (**Figure 3**) of the community structure (dis)similarities. The diversity analysis highlighted that, within the CV barn, all element protist communities differed (*p* ≤ 0.001) except between the house and stable fly. In the FT barn, protist species overlapped across component pairs except in comparison with the stable fly community. When comparing individual elements by management style, the CV and FT manure samples were significantly different, as were the CV and FT lagoon samples. When management systems were combined into components, all comparisons showed differences in beta diversity analyses. The effect sizes (pseudo-F, R²) indicate that many of the reported significant differences are driven primarily by communities dominated by a small number of taxa (**Table 1**).

**Table 1:** Protists diversity comparisons: the beta diversity (Bray-Curtis) indices are presented as comparisons between individual elements of the management (Mgt) systems, cross-vent (CV) and flow-through (FT), or as comparisons of the combined management-style components: manure (M), lagoon (L), trough (T), house fly (HF), and stable fly (SF).

Within Mgt system comparisons:					Between Mgt system comparisons:				
CV	CV	n	Statistic*	*p*-value	CV	FT	n	Statistic*	*p*-value
M	L	40	7.803	**0.001**	All	All	118	2.829	**0.001**
M	T	30	7.781	**0.001**	M	M	40	4.453	**0.001**
M	HF	25	5.192	**0.001**	L	L	40	6.580	**0.001**
M	SF	25	5.447	**0.001**	T	T	20	1.637	0.087
L	T	30	9.158	**0.001**	HF	HF	10	0.824	0.699
L	HF	25	6.961	**0.001**	SF	SF	8	2.369	0.019
L	SF	25	6.325	**0.001**					
T	HF	15	17.850	**0.001**					
T	SF	15	14.511	**0.001**					
HF	SF	10	2.831	0.019					
					Combined Mgt system comparisons:				
FT	FT	n	Statistic*	*p*-value	CV+FT	CV+FT	n	Statistic*	*p*-value
M	L	40	11.038	**0.001**	M	L	80	11.102	**0.001**
M	T	30	9.946	**0.001**	M	T	60	14.565	**0.001**
M	HF	25	4.412	**0.001**	M	HF	50	7.544	**0.001**
M	SF	23	2.323	0.017	M	SF	48	4.982	**0.001**
L	T	30	8.770	**0.001**	L	T	60	12.789	**0.001**
L	HF	25	8.034	**0.001**	L	HF	50	10.927	**0.001**
L	SF	23	2.926	0.002	L	SF	48	5.998	**0.001**
T	HF	15	9.094	**0.001**	T	HF	30	21.753	**0.001**
T	SF	13	3.124	0.013	T	SF	30	11.432	**0.001**
HF	SF	8	2.256	0.016	HF	SF	18	2.489	**0.001**

Significant *p*-values (p ≤ 0.001) are in bold.

*Pseudo F-statistic, calculated by dividing the variation between groups by the variation within the groups then multiplying by group numbers to derive the *p*-value.

### 3.2 Protist species within the dairy

The mean percent abundance of protists in each of the dairy elements, components, and management-style cohorts is provided (**Table 2**). No single protist species was present in every sample taken nor in every element sampled (CV and FT within each component). *Pseudoperonospora cubensis* was found in all except CV stable fly samples; therefore, it was found across all surveyed components when management systems were combined. *Pseudoperonospora cubensis* was the most prevalent protist in each management area. It was the only protist found in CV manure samples (percent relative abundance range: 0 – 100%). It was also present in FT manure samples (0 – 100%), CV lagoon samples (0 – 37.8%), FT lagoon samples (0 – 100%), CV house fly samples (0 -100%), and FT house fly samples (0 – 100%). *Paramecium biaurelia* was the most prevalent protist found in the CV trough samples (27.7 - 91.6%), FT stable fly samples (0 – 65.5%), and FT trough samples (0 – 87.8%). It was the second most prevalent protist in each management area. *Hammondia hammondi* was the most prevalent protist in the CV stable fly samples (0 – 100%) and the second most prevalent protist in CV house fly samples (0 - 100%) and FT house fly samples (0 – 47.5%). *Neobalantidium coli* (= Balantidium or Balantiodes) was the fourth most prevalent protist and was found in the wet environments of the CV through samples (0 – 66.7%) and FT trough samples (0 – 41.3%), in addition to CV lagoon samples (0 – 94.6%) and FT lagoon samples (0 – 96.4%). *Acanthamoeba* sp. was detected only in low abundance in the wet environment of the CV lagoon samples (0 – 76.8%). *Thalassiosira* sp. was detected in CV lagoon samples (0 – 76.8%), FT trough samples (0 – 54.8%), and FT stable fly samples (0 – 7.4%). *Reticulomyxa* sp. was found in CV trough samples (0 – 28.6%) and FT trough samples (0 – 48.3%). *Stylonychia lemnae* was detected in CV trough samples (0 – 6.1%) and FT trough samples (0 – 23.9%).

**Table 2:** The top *protists* in each of the dairy elements, components, and management-system cohorts and their relative abundance (%) in combined samples.

	Elements				Components:	
	%	CV	%	FT	%	CV + FT
MANURE	100.00	*Pseudoperonospora cubensis*	90.94	*Pseudoperonospora cubensis*	96.80	*Pseudoperonospora cubensis*
		* *	9.06	*Paramecium biaurelia*	3.20	*Paramecium biaurelia*
LAGOON	78.52	*Pseudoperonospora cubensis*	87.94	*Pseudoperonospora cubensis*	81.66	*Pseudoperonospora cubensis*
	9.32	*Acanthamoeba* sp.	12.06	*Neobalantidium coli*	7.96	*Neobalantidium coli*
	6.25	*Thalassiosira* sp.		* *	6.21	*Acanthamoeba* sp.
	5.91	*Neobalantidium coli*		* *	4.17	*Thalassiosira* sp.
TROUGH	69.66	*Paramecium biaurelia*	51.58	*Paramecium biaurelia*	61.09	*Paramecium biaurelia*
	14.90	*Pseudoperonospora cubensis*	23.86	*Pseudoperonospora cubensis*	19.15	*Pseudoperonospora cubensis*
	7.41	*Reticulomyxa filosa*	7.80	*Thalassiosira* sp.	6.51	*Reticulomyxa filosa*
	6.67	*Neobalantidium coli*	6.66	*Stylonychia lemnae*	5.69	*Neobalantidium coli*
	1.36	*Stylonychia lemnae*	5.51	*Reticulomyxa filosa*	3.87	*Stylonychia lemnae*
		* *	4.59	*Neobalantidium coli*	3.70	*Thalassiosira* sp.
HOUSE FLY	60.00	*Pseudoperonospora cubensis*	50.82	*Pseudoperonospora cubensis*	56.56	*Pseudoperonospora cubensis*
	40.00	*Hammondia hammondi*	49.18	*Hammondia hammondi*	43.44	*Hammondia hammondi*
STABLE FLY	100.00	*Hammondia hammondi*	65.46	*Paramecium biaurelia*	65.97	*Hammondia hammondi*
		* *	27.14	*Pseudoperonospora cubensis*	22.28	*Paramecium biaurelia*
		* *	7.40	*Thalassiosira* sp.	9.23	*Pseudoperonospora cubensis*
				* *	2.25	*Thalassiosira* sp.
	Management System:					
	%	CV	%	FT		
ALL COMPONENTS:	63.85	*Pseudoperonospora cubensis*	60.87	*Pseudoperonospora cubensis*		
	15.86	*Paramecium biaurelia*	21.63	*Paramecium biaurelia*		
	8.96	*Hammondia hammondi*	5.46	*Hammondia hammondi*		
	3.67	*Neobalantidium coli*	5.10	*Neobalantidium coli*		
	3.39	*Acanthamoeba* sp.	2.88	*Thalassiosira* sp.		
	2.28	*Thalassiosira* sp.	2.22	*Stylonychia lemnae*		
	1.69	*Reticulomyxa filosa*	1.84	*Reticulomyxa filosa*		
	0.31	*Stylonychia lemnae*				

### 3.3 Indicator species

Indicator protist species analyses of percent relative abundance by LEfSe were used to determine the taxa that best discriminated the composition between the different cohorts (**Supplementary Table 2**). Due to the small number of species identified at this dairy, these results could be biased toward overinterpretation and should be considered speculative. The analyses describe three main outputs comparing the differences in protist community among composition of 1) the CV and FT management areas; 2) the elements (CV manure, CV lagoon, CV trough, CV house fly, CV stable fly, FT manure, FT lagoon FT trough, FT house fly, and FT stable fly); and 3) the components (manure, lagoon, trough, house fly, and stable fly). The CV management barns were discriminated by *Pseudoperonospora cubensis* and *Thalassiosira* sp. No species discriminated the FT management barns. All elements of the CV barns had discriminatory species except manure and stable flies; the only FT element with discriminatory species was the trough.

### 3.4 Comparisons of species in cohorts

The overall comparison of protist species found in each dairy element is represented in **Figure 1**.

#### 3.4.1 Two-way comparisons of the management style by component

An assessment by management style of the number of species in each component was performed (**Table 3**). In total, eight protist species were identified from the samples. Seven (77.8%) species were shared between the CV and FT barns (*Pseudoperonospora cubensis*, *Paramecium biaurelia*, *Hammond hammondi*, *Neobalantidium coli*, *Thalassiosira* sp., *Stylonychia lemnae*, and *Reticulomyxa filosa*). *Acanthamoeba* sp. was unique to the CV barn. However, there were no species uniquely associated with CV manure, trough, house fly, or stable fly nor were there species uniquely associated with FT manure, lagoon, or house fly. The manure shared *Pseudoperonospora cubensis* between the two management systems. The lagoons shared *Pseudoperonospora cubensis* and *Neobalantidium coli*, and the house flies shared *Pseudoperonospora cubensis* and *Hammondia hammondi.* The troughs shared five species: *Paramecium biaurelia*, *Pseudoperonospora cubensis*, *Reticulomyxa filosa*, *Neobalantidium coli*, and *Stylonychia lemnae*. The stable fly did not share species between the CV and FT area; however, the FT stable fly sample size was smaller, as these flies were less prevalent on the sampling date.

**Table 3:** Two-way Venn comparisons of management systems.

Component	Unique to cross-vent	Shared	Unique to flow-through
ALL	1 (12.5)	7 (77.8)	0 (0.0)
MANURE	0 (0.0)	1 (50.0)	1 (50.0)
LAGOON	2 (50.0)	2 (50.0)	0 (0.0)
TROUGH	0 (0.0)	2 (33.3)	4 (66.7)
HOUSE FLY	0 (0.0)	2 (100.0)	0 (0.0)
STABLE FLY	1 (33.3)	0 (0.0)	2 (66.7)
FLY (House + Stable)	0 (0.0)	2 (50.0)	2 (50.0)

Comparisons of the number (percent) of unique and shared species of protists (n = 8) of each component using a two-way comparison associated with the collection of samples from areas in which the management styles of cross-vent (CV) or flow-through (FT) were implemented.

#### 3.4.2 Five-way comparisons of the elements

Comparisons of the number of protists in each element or component with management systems combined (dairy) were conducted (**Table 4**). The species unique to the CV management barn was *Acanthamoeba* sp. in lagoon samples. Evaluation of the components demonstrated that the trough (6), stable fly (4), and lagoon (4) carried the greatest number of protist species, whereas the manure and house fly carried the fewest, with only two species each. No species were common to all CV components, and only *Pseudoperonospora cubensis* was common to all FT components.

**Table 4:** Five-way Venn comparisons of management systems.

	Mgt System	Manure	Lagoon	Trough	House Fly	Stable fly	All components
Unique	CV	0 (0.0)	2 (22.2)	3 (33.3)	0 (0.0)	0 (0.0)	1 (1.3)
	FT	0 (0.0)	0 (0.0)	2 (28.6)	0 (0.0)	0 (0.0)	0 (0.0)
	Dairy	0 (0.0)	1 (11.1)	2 (22.2)	0 (0.0)	0 (0.0)	1 (11.1)
Total	CV	1	4	5	2	1	8
	FT	2	2	6	2	3	7
	Dairy	2	4	6	2	4	8

Comparisons of the number (percent within each management style) of unique and total protists (n = 8) using a five-way comparison associated with each component. Samples were collected from areas in which the management (Mgt) systems of cross-vent (CV) or flow-through (FT) were implemented, including unique species across combined cross-vent and flow-through (dairy) data. Also listed is the total number of species found in each component.

#### 3.4.3 Comparisons of components by paired sets

Pairwise comparisons of the number of protists within each component (combined management styles) were investigated (**Table 5**). The greatest number of species were shared between the lagoon and trough samples (*Pseudoperonospora cubensis*, *Neobalantidium coli*, and *Thalassiosira* sp.) and between the trough and stable fly samples (*Pseudoperonospora cubensis*, *Paramecium biaurelia*, and *Thalassiosira* sp.). In all other comparisons, one to two protist species were shared. Only two of the eight protist species (*Pseudoperonospora cubensis* and *Hammondia hammondi*) were shared by these two fly species, which represent different feeding behaviors and associated physiological structures and gut systems.

**Table 5:** Two-way venn comparisons of components.

Components		Comparison		
#1	#2	Unique to #1	Shared	Unique to #2
MANURE	LAGOON	1 (20.0)	1 (20.0)	3 (60.0)
	TROUGH	0 (0.0)	2 (33.3)	4 (66.7)
	HOUSE FLY	1 (33.3)	1 (33.3)	1 (33.3)
	STABLE FLY	0 (0.0)	2 (50.0)	2 (50.0)
LAGOON	TROUGH	1 (14.3)	3 (42.9)	3 (42.9)
	HOUSE FLY	3 (60.0)	1 (20.0)	1 (20.0)
	STABLE FLY	2 (33.3)	2 (33.3)	2 (33.3)
TROUGH	HOUSE FLY	5 (71.4)	1 (14.3)	1 (14.3)
	STABLE FLY	3 (42.9)	3 (42.9)	1 (14.3)
HOUSE FLY	STABLE FLY	0 (0.0)	2 (50.0)	2 (50.0)

Comparisons of the number (percent) of unique and shared protists (n = 8) using a pairwise comparison associated with each component sampled within the dairy. Species data were combined across cross-vent and flow-through management system areas.

## 4 Discussion

The protists of the gut microbiome of dairy cattle and their commercial products (i.e., milk, butter, and cheese) have been well studied; however, the environmental microbiome within production barns, including elements that may serve as reservoirs for beneficial or pathogenic organisms, remains largely unexplored. In this study, we examined the differences among protist microbiomes using shotgun metagenomics on samples from components (manure lagoon, trough, house flies, and stable flies) in dairy barns managed by two different free-stall systems. Our focus was to develop a greater understanding of the spatial location and abundance of protist communities at a commercial dairy and to assess how pathogenic species might be shared among interconnected components, thereby informing hypotheses about possible reservoirs and paths of dispersal for future studies. We also aimed to delineate protist communities carried by primary fly species at the dairy, which offered possible mechanisms for dissemination via two different Diptera feeding systems. It should be noted that findings in this exploratory study are hypothesis-generating and require validation through further studies, including multi-dairy comparisons, longitudinal studies, sampling of other important sites within the dairy, and viability-based approaches, before conclusions can be drawn regarding pathogenicity.

Although one of our goals was to identify the presence of possible pathogens in specific dairy niches and flies, this does not overshadow the vital beneficial ecological functions that protists serve. They play foundational roles in food webs, decomposition, nutrient cycling, predatory regulation of bacteria and fungi, and mutually beneficial symbiotic relationships (Esteban and Fenchel, 2021). In general, protists in dairies are most studied when they are associated with pathogenesis or play a beneficial role in the health and well-being of cattle, such as the rumen’s ciliated protists (Newbold et al., 2015; Yu et al., 2024; López-Novo et al., 2025). However, dairies offer many moist environmental locations where protists can not only survive but potentially multiply (Van Kessel et al., 2011). Even on well-managed, clean facilities such as the dairy in this study, wet protist habitats persist due to cooling sprinkler systems, multiple wash waterways, and cleaning systems. LeJeune et al. (2001b) evaluated 469 cattle water troughs and found that 248 (52.9%) contained detectable protists. All troughs were also positive for coliform counts, thus offering a source of nutrition for protists that feed on bacteria. Supporting this, protozoa from dairy lagoon wastewater have been shown to decrease the load of the pathogen *Escherichia coli* O157:H7 (Ravva et al., 2010), raising the possibility of planned microbiome interactions, for example, the enhancement of some species of protists that prey on bacteria to shape bacterial or fungal pathogen abundance.

The dry summer during which this dairy was sampled was characterized by erratic rainfall, high evaporation rates, and high temperatures, conditions that are unfavorable for protists; therefore, emphasis was placed on sampling components with moisture content. At this dairy, troughs, manure, and lagoons are interconnected within the facilities. Water in troughs was predominantly sourced from surrounding groundwater, which can serve as a direct source of protists; however, trough water may also be inadvertently contaminated by cattle usage and other activities within the barns (LeJeune et al., 2001b; Douglas and Pandey, 2025). Cattle are free-stalled and multiple cattle use the troughs, which may lead to an exchange of organisms and spillage that contributes to moist habitats (LeJeune et al., 2001a). Manure was manually collected via vacuum systems or manure scrapers and deposited into interconnected lagoons, which also received wastewater from wash channels.

At this dairy, overall richness and number of taxa were similar between cross-vent and flow-through management systems. While multiple samples were taken across the dairy, some samples were amalgamated to provide broader coverage, which limited spatial resolution analysis. Preference for wet environments is a common trait among protists and, accordingly, the greatest number of protist species at this dairy were associated with troughs and lagoons. When analyzing differences in specific components, differences in species composition and abundance were found in lagoons and manure, where community composition may be influenced by changes in the immediate environment and interactions with other microbes (Qiao et al., 2024). The manure environment did not appear to be as favorable a substrate for protist existence as the wetter environments of this dairy. While the diversity and abundance of protist populations in the rumen initially have a strong influence on the subsequent manure, they shift with animal age, diet, environmental exposure, and insect interactions (Yuste et al., 2019; Neupane et al., 2021; Estrada et al., 2024). The lack of protist diversity in manure is consistent with a previous study that showed that cattle and poultry manure applications can decrease soil protist community structure diversity (Nguyen et al., 2022).

*Pseudoperonospora cubensis* was found in all components when management systems were combined. It occurs worldwide and is an obligate plant parasite of many cucurbit species (Savory et al., 2011; Kikway et al., 2025). Its presence in components at the dairy is not surprising considering its widespread distribution and the use of culled vegetables and silage as a feed resource in cattle (Lalramhlimi et al., 2022). Pathogenicity of the protist was not determined since analysis by sequencing does not discriminate between live and dead organisms. It was the most abundant protist identified in manure, found in 42.5% of samples. The only other protist identified in manure was *Paramecium biaurelia*, found in 2.5% of samples. While this is not the typical freshwater habitat of *Paramecium*, the dairy ecosystem includes plenty of manure and wastewater runoff that contain bacteria and decomposing organic matter for this phagocytizing protist to consume (Van Houten, 2023). *Paramecium* species have been previously identified as associated with viscous scum that formed at the surface of wastewater lagoons (Ganapati and Amin, 1972). Although paramecia do not directly cause disease and, in fact, are beneficial, they can inadvertently assist in the transmission of harmful microorganisms. Adverse risk from the accidental transfer of endosymbionts and engulfed harmful prey by this paramecium is considered negligible (Peterson et al., 2013; Bella et al., 2016).

Lagoons are storage and treatment structures for manure. As in manure, the most abundant protist in lagoon samples was also *Pseudoperonospora cubensis*, found in 57.5% of samples. The second most abundant was the pathogen *Neobalantidium coli* (*Balantidium coli*), found in 5% of the lagoon samples. This organism is capable of infecting mammals, usually through oral ingestion of cysts in contaminated food or water, and may thrive asymptomatically in hosts (Schuster and Ramirez-Avila, 2008; Pomajbíková et al., 2013; Plutzer and Karanis, 2016). Problems arise in hosts experiencing malnutrition and immunosuppression (Palanivel et al., 2005; Plutzer and Karanis, 2016; Hassan et al., 2017). Animals such as insects may act as transport vectors in the dispersal of ciliates (Cochak et al., 2021). The presence of *Neobalantidium coli* was limited to lagoon and trough compartments at this dairy and was found only in low abundance. Lagoons and trough environments align with its preferred waterborne habitat (Ponce-Gordo and García-Rodríguez, 2021).

*Acanthamoeba* sp. was another protist found only in limited compartments, in 5% of the lagoon samples, and in low abundance. It is an environmentally ubiquitous protist that is highly adapted to surviving in a variety of environments, whether natural or artificial (Borecka et al., 2020). It is osmotolerant and thermotolerant and has been found in dairy environments, including in the milk used for calf feeding (Bahrami et al., 2024). *Acanthamoeba* feeds on bacteria, fungi, and algae via phagocytosis. It contributes greatly to the control of bacteria; however, these engulfed organisms can survive longer, multiply better, and, if pathogenic, can have higher virulence while living inside the amoebae (Vaerewijck et al., 2014; Kot et al., 2018; Samara et al., 2020; Wang et al., 2023). In previous studies, *Acanthamoeba* sp. in dairy slurries has been implicated in the persistence of the pathogen *Mycobacterium avium*, a progressive wasting disease in cattle (Greub and Raoult, 2004; White et al., 2010; Salgado et al., 2015; Samba-Louaka et al., 2018). However, the spread of engulfed harmful organisms by *Acanthamoeba* is usually a negligible risk to cattle (Bahrami et al., 2025). A diatom, *Thalassiosira* sp., was found in 2.5% of lagoon samples and represents an innocuous photosynthetic protist that is a primary producer essential for carbon cycling in aquatic environments (Stoermer and Julius, 2003).

Troughs harbored the highest variety of protist species, with the most prevalent species being *Paramecium biaurelia* (found in 89.5% of trough samples). *Paramecium* species have previously been found in cattle water troughs (Schmidt, 2015). The second most abundant species in the troughs was *Pseudoperonospora cubensisa*, found in 100% of trough samples but at lower relative abundance than *Paramecium biaurelia*. The third most abundant was *Reticulomyxa filosaa*, found in 42.1% of trough samples. *Reticulomyxa filosa* is a foraminifera common to freshwater, moist environments, decomposing matter, and damp soils (Pawlowski et al., 1999). This basically describes much of the dairy environment, which could easily make a quality habitat for a foraminifera. This foraminifera feeds on microbes and larger organisms by engulfing and phagocytizing their prey. *Neobalantidium coli* was the fourth most abundant taxon in troughs, identified in 10.5% of samples. *Stylonychia lemnae*, found exclusively in troughs (47.4% of samples), was the fifth most abundant species. It is an innocuous ciliate globally found in freshwater and soil (Schmidt et al., 2006; Berger, 2012). The least abundant taxon was the diatom *Thalassiosira* sp., found in 31.6% of trough samples but only in FT troughs.

Insects represent a possible reservoir and another mechanism for the spread of protists. Protists may be parasitic and infect insects or be phoretic and use insects to transport their oocysts mechanically or biologically (Bessette and Williams, 2022; Mondal et al., 2023). House fly larval grazing on dairy manure can also reshape microbial communities and increase protist diversity in cattle manure (Neupane et al., 2021) The two fly species collected in this study harbored a different diversity of protists. Filth fly microbiota is strongly influenced by the behavioral habits of the particular fly species; thus, two species representing different feeding systems (house flies and stable flies) were sampled. The house fly carried two protist species, with the most abundant being *Pseudoperonospora cubensis* (found in 50% of samples), followed by *Hammondia hammondi* (found in 40% of house fly samples). The stable fly carried five protist species, with the most abundant being *Hammondia hammondi* (found in 25% of samples). The stable fly represents a hematophagous species, which are highly mobile as adults. Stable flies tend to blood feed once per day and then rest in the shade at on-farm locations as opposed to resting on cattle (Hogsette et al., 1989; Showler and Osbrink, 2015). These flies are therefore exposed to many dairy locations, such as loafing sites, holding pens, feeding areas, and milking parlors, near the cattle that support them (Hogsette et al., 1989). In contrast, house flies are omnivorous sponge feeders, acquiring food from many sources. They move in short, fragmented, meandering flights seeking wet and decaying organic matter including manure, bedding, feed, milking barns, stalls, and a multitude of other sites within dairies (Gerry et al., 2011; Geden et al., 2021). Both species had ample opportunity to interact with the targeted environmental samples collected in this study.

Of the protists found, *Hammondia hammondi*, an obligate alveolate parasite, was exclusively carried by the flies and not found in manure, trough, or lagoon samples. It is closely related to *Toxoplasma gondii* but infects a smaller group of hosts (felids, canids, small rodents, caprids, and swine) and is considered non-pathogenic (Dubey et al., 2002). Its final host is a domestic feline (Frenkel and Dubey, 1975; Wong et al., 2020). Domestic felines are commonly found on dairies as valued allies for pest control, in addition to serving as companions to dairy workers (Crawford et al., 2025). *Hammondia* is not known to cause disease in cattle (Santos et al., 2010).

Whereas the plant pathogen *Pseudoperonospora cubensis* was found in all components sampled at the dairy, other protists were more localized. The alveolate parasite *Hammondia hammondi* was limited to flies. The amoeba *Acanthamoeba* sp., a foraminifer, *Reticulomyxa filosa*, and possibly the pathogenic ciliate *Neobalantidium coli* were identified in the wet environments of the lagoons and troughs. The paramecium *Paramecium biaurelia* was identified in manure, troughs, and stable fly samples. While we are reporting the presence of organisms that could be potential causes of pathogenesis, it should be noted that there is no causal data from this dairy directly tying any adverse health outcomes to a protist contagion. Metagenomic DNA sequence data cannot distinguish viable oocysts from DNA fragments, and clinical outcomes were not measured in this study.

Dairy health data were summarized for the month prior to sampling, the sampling month, and the month after the samples were taken from the dairy. Data are presented as a percent of the herd affected during those times: 0.31%, 0.29%, and 0.64% cattle died; 0.34%, 0.52%, and 0.72% retained placentas; and 1.00%, 0.93%, and 0.93% abortions occurred, respectively. Additionally, the following cases were reported: 6.31%, 7.00%, and 7.41% mastitis; 0.36%, 0.36%, and 0.33% metritis; 0.52%, 0.38%, and 0.47% pneumonia; 0.24%, 0.17%, and 0.38% ketosis; 0.12%, 0.17%, and 0.19% abomasal displacement; 0.02%, 0.03%, and 0.02% hemorrhagic bowel disease; and 0.10%, 0.02%, and 0.19% fever. These health records provide an overall view of the issues occurring in relation to the presence of known protists in different components of the barns. We recognize and strongly advocate that the mere presence of a known pathogen does not translate directly into pathology. An infection would depend on a multitude of factors, starting with the vitality of the organism identified by DNA analysis and the likelihood of contact with a transmissible load from the environmental elements. In addition, the cattle’s general health and immunocompetency would influence morbidity or mortality. Cattle at this dairy received a medication regimen against infectious bovine respiratory diseases, parainfluenza, coronavirus, leptospirosis, blackleg, enterotoxemia, mastitis, scours, viral diarrhea, and pinkeye. While possible pathogenic protists’ DNA was identified at this dairy, there was a low incidence of disease and other complications recorded in the health records. This is likely attributed to the management’s meticulous health and sanitation program. Infection is always dependent on the pathogen load and the health status of the cattle.

## 5 Conclusion

In this study, our focus was to develop a greater understanding of the spatial location and abundance of protist communities at a working dairy and to compare the two management systems being implemented. Additionally, we aimed to observe how pathogenic species might be shared among the interconnected components, namely, troughs, manure, and lagoons, which could help hypothesize possible reservoirs and pathways of dispersal for future studies. Lastly, we sought to delineate the protist communities carried by the primary fly species at the dairy, which may represent mechanisms for dissemination via two different Diptera feeding systems. Overall, it is difficult to generalize about the two management systems since this was a single-dairy study and the number of protists found was low. Given this limitation, there were significant differences in the protist community composition among dairy components. Troughs and lagoons exhibited the highest diversity and stood out as harboring sites for protists. They had two possible carriers of pathogens, an amoeba and a ciliate, in addition to a possible plant pathogen and an animal pathogen. The lowest diversity was found in manure. Notably, stable flies carried more protist taxa than house flies. Both fly species, the generalized sponge feeders and the specialized blood feeders, carried an obligate intracellular alveolate parasite and a plant pathogen. Stable flies also carried innocuous diatoms and paramecia. There was a lack of overlapping protist profiles between the house flies and the other dairy elements surveyed, suggesting minimal sharing of microorganisms across these components, whereas stable flies overlapped with three species in the trough samples.

These results provide an initial framework for assessing management systems and identifying specific sites for potential protist control or enhancement efforts. The conclusions in this study are limited by the fact that only a single dairy was sampled at a single time point. However, this dairy offered the ability to limit confounding factors, such as water, feed, equipment, cattle health regimen, and personnel, which were the same for both systems. Only DNA-based analysis was conducted, which does not differentiate between live and dead organisms, and no microscopy or targeted PCR was performed. Further studies are needed to include multi-dairy comparisons and sampling of other important sites within the dairy, such as cattle, milking parlors, silage, and feed. Longitudinal studies would be required to assess the seasonality of the occurrence of these protists and the impact of abiotic compounding factors, such as farm management practices. While this study provides a glimpse at possible protist locations, as measured by DNA detection, the efficacy of mitigation management applied to manipulate these communities, such as lagoon aeration and waste separation techniques, facilities cleaning schedules, manure handling, and direct control measures, remain to be evaluated. More studies are required before these findings can be translated into broader management recommendations.

## Data Availability

The datasets presented in this study can be found in online repositories. The names of the repository/repositories and accession number(s) can be found in the article/**Supplementary Material**.
