# Supplementary material for: Protist community sites and structure under two barn management systems at a commercial dairy
Source: Front Microbiomes. 2026 May 14;5:1803341. doi: 10.3389/frmbi.2026.1803341 (PMC13217480; doi:10.3389/frmbi.2026.1803341)
Supplement: Supplementary file 1 [file DataSheet1.pdf]

Table S1. Protists found within the specified single or shared by the collective components of combined cross-vent and flow-through free stall microbiome results.

Components

MANURE	LAGOON	TROUGH	HOUSE FLY	STABLE FLY
2	4	6	2	5
Paramecium biaurelia<br>*Pseudoperonospora cubensis*	*Acanthamoeba sp*<br>*Neobalantidium coli*<br>*Pseudoperonospora cubensis*<br>*Thalassiosira sp*	*Neobalantidium coli*<br>*Paramecium biaurelia*<br>*Pseudoperonospora cubensis*<br>*Reticulomyxa filosa*<br>*Stylonychia lemnae*<br>*Thalassiosira sp*	*Hammondia hammondi*<br>*Pseudoperonospora cubensis*	*Hammondia hammondi*<br>*Paramecium biaurelia*<br>*Pseudoperonospora cubensis*<br>*Thalassiosira sp*

2-way

MANURE<br>LAGOON	MANURE<br>TROUGH	MANURE<br>HOUSE FLY	MANURE<br>STABLE FLY	LAGOON<br>TROUGH	LAGOON<br>HOUSE FLY	LAGOON<br>STABLE FLY	TROUGH<br>HOUSE FLY	TROUGH<br>STABLE FLY	HOUSE FLY<br>STABLE FLY
1	2	1	2	3		1	1	3	2
Pseudoperonospora cubensis	*Paramecium biaurelia*<br>*Pseudoperonospora cubensis*	*Pseudoperonospora cubensis*	*Paramecium biaurelia*<br>*Pseudoperonospora cubensis*	*Neobalantidium coli*<br>*Pseudoperonospora cubensis*<br>*Thalassiosira sp*		*Pseudoperonospora cubensis*	*Pseudoperonospora cubensis*	*Paramecium biaurelia*<br>*Pseudoperonospora cubensis*<br>*Thalassiosira sp*	*Hammondia hammondi*<br>*Pseudoperonospora cubensis*

3-way

MANURE<br>LAGOON<br>TROUGH	MANURE<br>LAGOON<br>HOUSE FLY	MANURE<br>LAGOON<br>STABLE FLY	MANURE<br>TROUGH<br>HOUSE FLY	MANURE<br>TROUGH<br>STABLE FLY	MANURE<br>HOUSE FLY<br>STABLE FLY	LAGOON<br>TROUGH<br>HOUSE FLY	LAGOON<br>TROUGH<br>STABLE FLY	LAGOON<br>HOUSE FLY<br>STABLE FLY	TROUGH<br>HOUSE FLY<br>STABLE FLY
1	1	1	1	2	1	1	2	1	1
Pseudoperonospora cubensis	*Pseudoperonospora cubensis*	*Pseudoperonospora cubensis*	*Pseudoperonospora cubensis*	*Paramecium biaurelia*<br>*Pseudoperonospora cubensis*	*Pseudoperonospora cubensis*	*Pseudoperonospora cubensis*	*Pseudoperonospora cubensis*<br>*Thalassiosira sp*	*Pseudoperonospora cubensis*	*Pseudoperonospora cubensis*

4-way

MANURE<br>LAGOON<br>TROUGH<br>HOUSE FLY	MANURE<br>LAGOON<br>TROUGH<br>STABLE FLY	MANURE<br>LAGOON<br>HOUSE FLY<br>STABLE FLY	MANURE<br>TROUGH<br>HOUSE FLY<br>STABLE FLY	LAGOON<br>TROUGH<br>HOUSE FLY<br>STABLE FLY
1	1	1	1	1
Pseudoperonospora cubensis	*Pseudoperonospora cubensis*	*Pseudoperonospora cubensis*	*Pseudoperonospora cubensis*	*Pseudoperonospora cubensis*

5-way

MANURE<br>LAGOON<br>TROUGH<br>HOUSE FLY<br>STABLE FLY
1
Pseudoperonospora cubensis
