# Supplementary material for: Protist community sites and structure under two barn management systems at a commercial dairy
Source: Front Microbiomes. 2026 May 14;5:1803341. doi: 10.3389/frmbi.2026.1803341 (PMC13217480; doi:10.3389/frmbi.2026.1803341)
Supplement: Supplementary file 2 [file DataSheet2.pdf]

Table S2. The top protists in each of the dairy elements, components, and management style cohorts and their percent abundance (%) of combined samples.

ELEMENTS:	%	MANURE	%	LAGOON	%	TROUGH	%	HOUSE FLY	%	STABLE FLY
Cross vent	100.00	*Pseudoperonospora cubensis*	78.52	*Pseudoperonospora cubensis*	69.66	*Paramecium biaurelia*	60.00	*Pseudoperonospora cubensis*	100.00	*Hammondia hammondi*
			9.32	*Acanthamoeba sp*	14.90	*Pseudoperonospora cubensis*	40.00	*Hammondia hammondi*		
			6.25	*Thalassiosira sp*	7.41	*Reticulomyxa filosa*				
			5.91	*Neobalantidium coli*	6.67	*Neobalantidium coli*				
					1.36	*Stylonychia lemnae*				
Flow through	90.94	*Pseudoperonospora cubensis*	87.94	*Pseudoperonospora cubensis*	51.58	*Paramecium biaurelia*	50.82	*Pseudoperonospora cubensis*	65.46	*Paramecium biaurelia*
	9.06	*Paramecium biaurelia*	12.06	*Neobalantidium coli*	23.86	*Pseudoperonospora cubensis*	49.18	*Hammondia hammondi*	27.14	*Pseudoperonospora cubensis*
					7.80	*Thalassiosira sp*			7.40	*Thalassiosira sp*
					6.66	*Stylonychia lemnae*				
					5.51	*Reticulomyxa filosa*				
					4.59	*Neobalantidium coli*				

COMPONENTS:	%	MANURE	%	LAGOON	%	TROUGH	%	HOUSE FLY	%	STABLE FLY
Cross vent + Flow through	96.80	*Pseudoperonospora cubensis*	81.66	*Pseudoperonospora cubensis*	61.09	*Paramecium biaurelia*	56.56	*Pseudoperonospora cubensis*	65.97	*Hammondia hammondi*
	3.20	*Paramecium biaurelia*	7.96	*Neobalantidium coli*	19.15	*Pseudoperonospora cubensis*	43.44	*Hammondia hammondi*	22.28	*Paramecium biaurelia*
			6.21	*Acanthamoeba sp*	6.51	*Reticulomyxa filosa*			9.23	*Pseudoperonospora cubensis*
			4.17	*Thalassiosira sp*	5.69	*Neobalantidium coli*			2.52	*Thalassiosira sp*
					3.87	*Stylonychia lemnae*				
					3.70	*Thalassiosira sp*				

MANGEMENT STYLES:	%	Cross Vent	%	Flow Through
Combined Components	63.85	*Pseudoperonospora cubensis*	60.87	*Pseudoperonospora cubensis*
	15.85	*Paramecium biaurelia*	21.63	*Paramecium biaurelia*
	8.96	*Hammondia hammondi*	5.46	*Hammondia hammondi*
	3.67	*Neobalantidium coli*	5.10	*Neobalantidium coli*
	3.39	*Acanthamoeba sp*	2.88	*Thalassiosira sp*
	2.28	*Thalassiosira sp*	2.22	*Stylonychia lemnae*
	1.69	*Reticulomyxa filosa*	1.84	*Reticulomyxa filosa*
	0.31	*Stylonychia lemnae*		
