# Supplementary material for: Protist community sites and structure under two barn management systems at a commercial dairy
Source: Front Microbiomes. 2026 May 14;5:1803341. doi: 10.3389/frmbi.2026.1803341 (PMC13217480; doi:10.3389/frmbi.2026.1803341)
Supplement: Supplementary file 3 [file DataSheet3.pdf]

Supplementary Material

2 MATERIALS & METHODS

2.3 Sequencing and bioinformatics analyses

Pseudoperonospora cubensis are known as water mold, however this species does not classify as a fungus. They are described as a “fungus-like” protist (Savory et al. 2011; Piepenbring 2015). As such they were included in this analysis as a protist per the reference database used by CosmosID taxonomic profiling. Although there seems to be some debate as to whether they are protists Straminipilas or their own distinct group related to diatoms or algae, the taxonomy used for this study was as follows: Eukaryota, Clade: Sar, Clade: Straminipila, Class: Oomycetes, Order: Peronosporales, Family: Peronosporaceae, Genus: *Pseudoperonospora*) (Thines and Choi 2016).

Data Availability Statement: The datasets generated and/or analyzed during the current study are available at https://www.ncbi.nlm.nih.gov. NCBI BioProject PRJNA948024.
